# Upper partial sternal split for pediatric cardiac surgery

**DOI:** 10.1007/s11748-023-01996-7

**Published:** 2024-01-16

**Authors:** Fumiaki Shikata, Jay Shah, Supreet Marathe, Jessica Suna, Nelson Alphonso, Prem Venugopal

**Affiliations:** 1 Queensland Pediatric Cardiac Service, Queensland Children’s Hospital, Level 7F, Clinical Directorate, PO Box 3474, South Brisbane, QLD 4101 Australia; 2 School of Clinical Medicine, Children’s Health Queensland Clinical Unit, University of Queensland, Brisbane, QLD Australia; 3 Queensland Pediatric Cardiac Research, Queensland Children’s Hospital, South Brisbane, QLD Australia; 4 University of Queensland, Brisbane, QLD Australia; 5 Ochsner Medical Center, New Orleans, LA USA

**Keywords:** Minimally invasive surgery, Congenital heart surgery, Outcomes, Congenital heart disease

## Abstract

**Objectives:**

We introduced the use of an upper partial sternal split for pediatric cardiac surgical procedures in our unit in 2016. We report the outcomes of our experience in 51 patients using this approach.

**Methods:**

From February 2016 to September 2022, 51 patients underwent congenital cardiac surgical procedures using an upper partial sternal split including vascular ring repair (*n* = 20), subaortic membrane (*n* = 12), ventricular septal defect closure with aortic valve resuspension (*n* = 9), aortic arch repair (*n* = 4), pulmonary artery band (*n* = 2), pulmonary artery sling (*n* = 1), supravalvular aortic stenosis (*n* = 1), aortic valve replacement (*n* = 1), and pulmonary artery plasty (*n* = 1). The surgical approach involved a midline skin incision, based on the manubrium, followed by an upper manubriotomy. No special surgical instrumentation was required. Median patient age was 2.9 years (IQR 1.3, 6.0); median body weight was 15 kg (IQR 9.8, 20).

**Results:**

There was no mortality and no patient required intraoperative conversion to full sternotomy. One patient required re-exploration for bleeding when the incision was converted to a full sternotomy. There were no wound complications in any patient. Twenty-one patients (41%) were extubated on the table and of the remaining 30 patients, 23 patients (76%) were extubated within 24 h of surgery. Eleven patients did not require intensive care unit (ICU) admission. Median ICU and hospital stay was 1 day (IQR 1, 1.25) and 5 days (IQR 4, 8) ,respectively.

**Conclusion:**

An upper partial sternal split approach is straightforward and can be performed safely with a preferable cosmetic result in selected pediatric cardiac operations.

**Supplementary Information:**

The online version contains supplementary material available at 10.1007/s11748-023-01996-7.

## Introduction

Minimally invasive cardiac surgery (MICS) for adult cardiac surgery has become increasingly popular due to advances in surgical instrumentation, wound retractors, cannulas and cannulation techniques, and myocardial protection. This allows surgeons to perform procedures using video-assisted thoracoscopy or through small incisions under direct vision [1]. Reported benefits include shorter hospital stay, less postoperative pain, as well as an improved cosmetic appearance [2]. MICS procedures for pediatric patients have been increasingly adopted since the 2000s, especially for managing simple congenital heart defects, such as atrial septal defects (ASD) and ventricular septal defects (VSD) using partial lower sternal splits or thoracotomies [3]. However, existing techniques are not well suited for managing aortic arch anomalies and aortic valve lesions, due to the limited exposure of the aortic arch and left ventricular outflow tract and potentially suboptimal surgical outcomes. The upper partial sternal split approach for the management of congenital heart defects was initially reported by Miyaji et al. who described the outcomes of subaortic membrane resection procedures using video-assisted thoracoscopic surgery (VATS) [4].

Our interest in developing this approach was triggered by an increase in the number of requests from parents and patients for smaller skin incisions. Consequently, one surgeon in the unit began using the upper partial sternal split approach at Queensland Children’s Hospital since 2016.

The aim of our study was to describe our technique for an upper partial sternal split in congenital heart surgery and report the outcomes of our initial experience with this approach.

## Methods

All patients under 18 years old who underwent congenital heart surgery using an upper partial sternal split at the Queensland Children’s Hospital from February 2016 to September 2022 were included in this study. Median patient age was 2.9 years (IQR 1.3,6.0) and median body weight was 15 kg (IQR 9.8,20). Operations without the use of cardiopulmonary bypass (CPB) included repair of vascular ring with reimplantation of aberrant subclavian artery and repair of double aortic arch. Those involving the use of CPB included resection of subaortic membrane, repair of distal hypoplastic aortic arch/coarctation, and closure of a subarterial VSD with aortic valve resuspension. The study was deemed a low and negligible risk project and the need for informed consent was waived by our institutional ethics committee.

## Operative techniques

### Upper partial sternal split

Through a manubrium based, limited, midline skin incision (approximately 3–5 cm), an upper manubriotomy is performed with an oscillating sternal saw. We did not need an additional transverse sternal cut at the inferior end of the partial sternotomy. Fixation sutures taken through the lower edges of the divided manubrium are used to stabilize the sternal retractor. A second single blade sternal retractor is placed to widen the surgical field vertically. After a subtotal thymectomy, the innominate vein is dissected and retracted with a silastic tape. We do not use any special surgical instrumentation for this approach. Video 1 demonstrates a subaortic membrane resection through an upper partial sternal split.

### Repair of a vascular ring

The commonest types of vascular rings we have operated include right aortic arch with a retro-esophageal aberrant left subclavian artery (SCA), Kommerell diverticulum, and a double aortic arch [5]. A pre-operative bronchoscopy is performed on table after induction of anesthesia if the pre-operative CT suggests compression of the trachea and bronchus by the vascular ring. Insertion of a nasogastric tube helps to identify the esophagus and an arterial line is placed in the left upper limb to confirm the patency of the reimplanted aberrant SCA after the procedure. Through an upper partial sternum split, the ascending aorta, proximal descending aorta, and all the neck vessels [including the left and right common carotid arteries (CCA), right subclavian artery and aberrant left SCA] are dissected. The ligamentum arteriosum is divided. aorta. A curved vascular clamp is applied just below the origin of the left SCA, and the distal end is temporary occluded using loosely applied titanium liga clips. The artery is divided, leaving enough length for closure of the aortic origin to avoid stenosis of the descending aorta. The SCA is dissected along its retro-esophageal course. It is then brought anterior to the trachea and esophagus on the left side. A side-biting clamp is placed across the left CCA. Near-infrared spectroscopy (NIRS) monitoring is used to confirm unimpaired brain perfusion. The left SCA is anastomosed to the left CCA in an end-to-side fashion with a running polypropylene suture. Patency of the anastomosis is assessed from pulse waves in the arterial tracing of left upper limb arterial monitoring line. Relief of tracheal compression is assessed by flexible bronchoscopy on table. An aortopexy to the under surface of the manubrium is performed if required.

### Cardiopulmonary bypass

After systemic heparinization, CPB is instituted by cannulating the ascending aorta and right atrium with a DLP one-piece arterial cannula and a DLP metal tip single venous cannula (Medtronic, Minnesota, USA), respectively.

### Subaortic membrane resection

The patient is maintained at normothermia during CPB. The left heart is vented through the main pulmonary artery. The aorta is cross clamped and cardioplegia is delivered antegrade into the ascending aorta. An oblique aortotomy is made into the non-coronary sinus. Stay sutures are placed above each aortic commissure to bring the aortic valve and the subaortic area into view. A flat malleable retractor is used to retract the valve leaflets. The fibromuscular shelf is resected *en bloc* and a myectomy is performed below the left half of the right coronary cusp. Video 1 demonstrates subaortic membrane resection through a partial sternal split.

### Closure of subarterial ventricular septal defect with aortic valve resuspension

Using a similar approach as for subaortic membrane resection, the VSD is exposed through an oblique aortotomy and closed directly with three pledgeted polypropylene sutures in such a way that the right coronary cusp is resuspended at the same time (Yacoub technique) [6].

### Repair of distal hypoplastic aortic arch/coarctation on beating heart

**Fig. 1:**
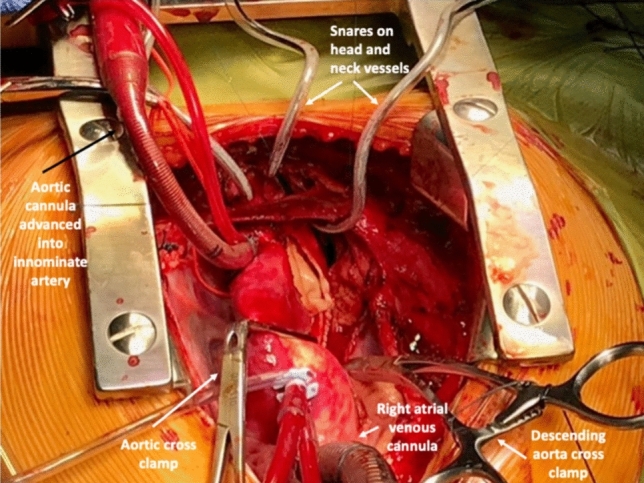
Set-up for arch repair without cardioplegic arrest through an upper partial sternal split

After initiation of CPB, the patient is cooled to 32 °C. The ascending aorta, innominate artery, left CCA, left SCA, descending thoracic aorta, and the ductus arteriosus are dissected. The ductus is ligated and divided. Titanium ligaclips are used to temporarily occlude the left CCA and left SCA. The distal aortic arch beyond the left SCA is ligated with a silk suture and divided. A cross clamp is placed across the descending thoracic aorta. At 32 °C, selective antegrade cerebral perfusion is commenced by maneuvering the arterial cannula into the innominate artery and snaring it. The juxta ductal segment is resected. A cardioplegia cannula is placed in the aortic root. Another cross clamp is placed beyond the cardioplegia cannula, and the heart is perfused with blood. An incision is made on the underside of the proximal aortic arch. The descending thoracic aorta is anastomosed to this opening in an end-to-side manner with continuous 6–0 polypropylene sutures. The arch is de-aired through the cardioplegia cannula, and the clamps are released. The set up for this operation is depicted in Fig. 1.

### Drain insertion and postoperative pain management

**Fig. 2:**
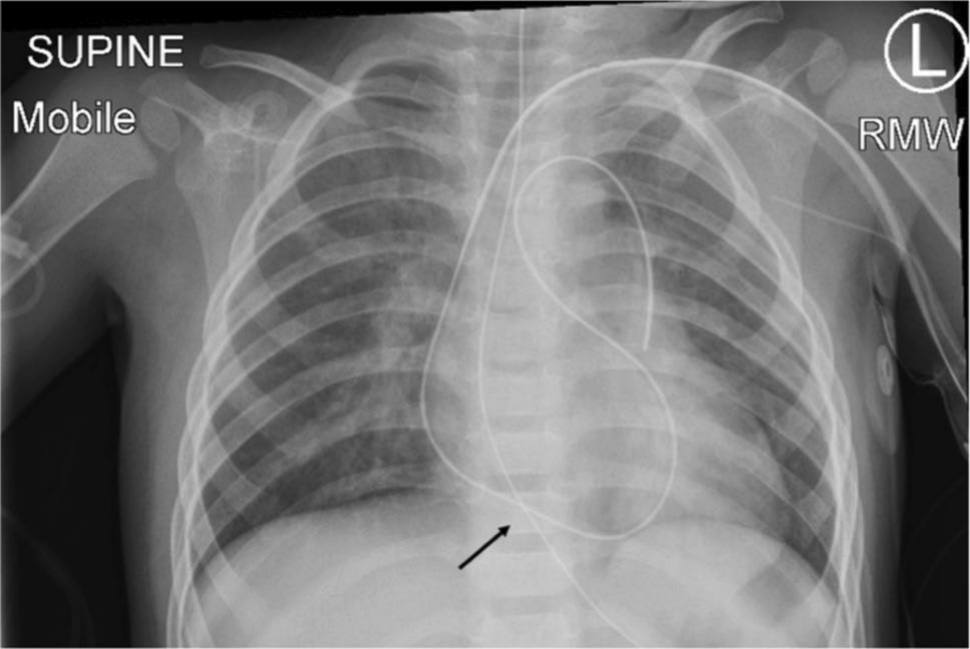
Chest X-ray showing loop of non-luminal channeled drain ensuring drainage of retro-cardiac space (black arrow)

**Fig. 3:**
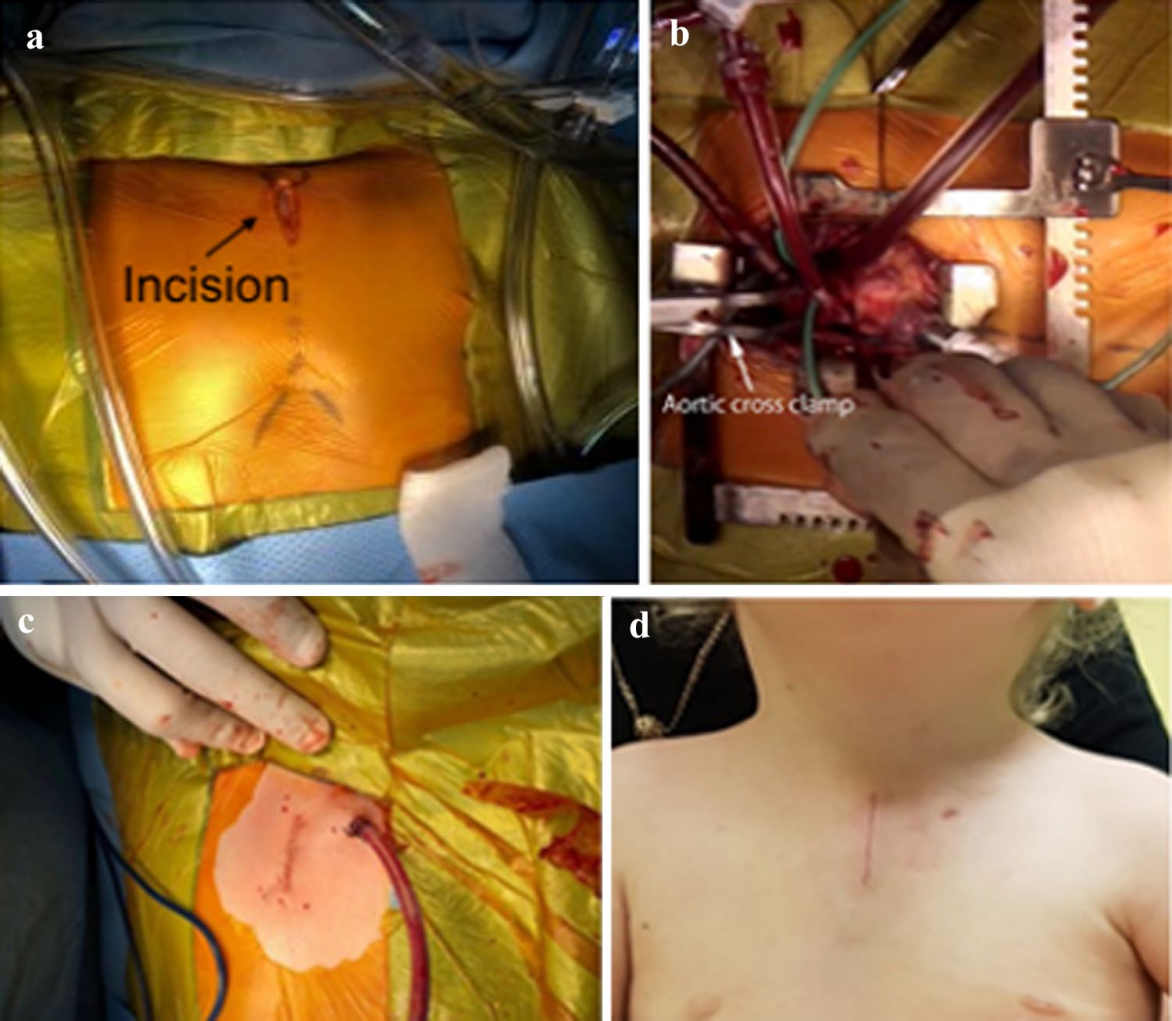
Wound appearance after upper partial sternal split. **a** Incision for upper partial sternotomy. **b** Operative view of subaortic membrane resection. **c** After skin closure with drain exiting through upper part of wound. **d** 1-month post-surgery

Bilateral intercostal nerve blocks are administered parasternally using injections of levobupivacaine prior to skin incision. A non-luminal channeled drain (Blake® drain, Ethicon, Inc, Somerville, NJ) is introduced from the left side of the incision into the mediastinum and/or pleural cavities (if opened) in every patient. Following the development of a pericardial effusion in a few patients, we modified our drain insertion technique in 2019 to ensure that a loop of the drain drains the retro-cardiac space (Fig. 2). The sternum is approximated with delayed absorbable sutures. After skin closure, On-Q PainBuster soaker catheters (I-Flow Corporation, Lake Forest, CA) are placed bilaterally into the subcutaneous tissues parallel to the sternotomy incision. The elastomeric pump is filled with levobupivacaine and connected to the PainBuster catheters for continuous infusion. Dexmedetomidine, patient-controlled analgesia with morphine or fentanyl, and oral paracetamol are administered postoperatively by a dedicated Acute Pain Service Team. The PainBuster systems are removed 2 days after surgery. The postoperative wound appearance is depicted in Fig. 3.

## Results

Between February 2016 and September 2022, the upper partial sternal split approach was used in 51 patients. Procedures included vascular ring repair (*n* = 20), subaortic membrane (*n* = 12), ventricular septal defect closure with aortic valve resuspension (*n* = 9), aortic arch repair (*n* = 4), pulmonary artery band (*n* = 2), pulmonary artery sling (*n* = 1), supravalvular aortic stenosis (*n* = 1), aortic valve replacement (*n* = 1), and pulmonary artery plasty (*n* = 1). The proportion of patients in whom the upper partial sternal split was successfully used during the study is given in Table 1.

**Table 1:** Proportion of patients undergoing the partial upper sternal split between February 2016 and September 2022

Procedure	Total number of procedures during study periods	Procedures performed through upper partial sternal split
		(*n*, %)
Vascular ring	41	20 (49%)
Subaortic membrane excision	43	12 (28%)
VSD closure + aortic valve resuspension	32	9 (28%)
Aortic arch repair	49	4 (8%)
Pulmonary artery banding	43	2 (5%)
Aortic valve replacement	9	1 (11%)
Supravalvular aortic stenosis repair	12	1 (8%)
Pulmonary artery sling	2	1 (50%)
Pulmonary artery reconstruction	16	1 (6%)

The median comprehensive Aristotle complexity score was 7.8 (IQR 6.5, 9.5). The median age was 2.9 years (IQR 1.3, 6.0) while the median weight was 15 kg (IQR 9.8, 20). Details of these 51 patients are presented in Table 2.

**Table 2:** Procedures performed through an upper partial sternal split

Characteristic	Patients *n* = 51
Age (years), median (IQR)	2.9 (1.3–6.0)
Weight (kg), median (IQR)	15 (9.8–20)
Comprehensive Aristotle score, median (IQR)	7.8 (6.5–9.5)
Procedure, *n* (%)	
Vascular ring	20 (39%)
Subaortic membrane excision	12 (23%)
VSD closure + aortic valve resuspension	9 (18%)
Aortic arch repair	4 (8%)
Pulmonary artery banding	2 (4%)
Aortic valve replacement	1 (2%)
Supravalvular aortic stenosis repair	1 (2%)
Pulmonary artery sling	1 (2%)
Pulmonary artery reconstruction	1 (2%)
CPB time (minutes), median (IQR) (*n* = 29)	38 (32–47)
ICU stay (days), median (IQR) (*n* = 40)	1 (1–1.25)
Hospital stay (days), median (IQR)	5 (4–8)
Ventilation time (hours), median (IQR) (*n* = 30)	8.5 (3–116)

*CPB* cardiopulmonary bypass, *ICU* intensive care unit, *IQR* interquartile range, *VSD* ventricular septal defect

Twenty-one patients (41%) were extubated on the table and of the remaining 30 patients, 23 patients (76%) were extubated within 24 h of surgery. For these 30 patients who were intubated on transfer to the intensive care unit (ICU), the median ventilation time was 8.5 h (IQR 3, 16). Eleven patients did not require ICU admission. The median ICU and hospital stay was 1 day (IQR 1, 1.25) and 5 days (IQR 4, 8), respectively.

There were no wound complications in any patient and no patient needed intraoperative conversion to a full sternotomy. One patient who underwent aortic valve replacement required re-exploration for bleeding during when the incision was converted to a full sternotomy. Three patients (8%) needed drainage of pericardial effusion out of which two were accomplished using needle pericardiocentesis while one patient needed surgical drainage. After revising the technique of pericardial drain placement in February 2019, no patient has required drainage of a pericardial effusion.

## Discussion

MICS techniques in adult cardiac surgery cannot be easily implemented in children as peripheral vessels are often too small to be cannulated. A lower partial sternal split for simple congenital heart defects in children, with or without transmediastinal cannulation, is a well-established approach. A previous report has demonstrated a return to school and engagement in high-intensity activities such as gymnastics earlier than after a conventional median sternotomy approach [7]. In addition, MICS in children can potentially reduce not only the physical trauma related to surgery, but also the emotional stress caused by a large skin scar and potential breast deformities, especially in pubertal girls [8]. A transverse sternal split and thoracotomy approach has been previously reported for pediatric cardiac cases [9, 10]. However, this method requires peripheral cannulation and concerns remain about distal perfusion of the cervical or femoral vessels.

We introduced the upper partial sternal split approach in selected pediatric cardiac operations at QCH in 2016, and gradually extended its application to more complex lesions, including repair of hypoplastic aortic arch. One additional benefit of our approach is that it does not require special instruments or peripheral cannulation, in contrast to other minimally invasive approaches described in children [5, 8, 9]. It is important to note that regarding the exposure of a target treatment zone of the procedures, the surgical view obtained from upper partial sternal split is same as that of conventional full sternotomy. Lower structures of the heart, especially the inferior vena cava, are not easily accessible through the upper partial sternal split approach but these parts are of no particular interest for the patients in whom an upper partial split is utilized. Nonetheless, the upper partial sternal split can easily be converted to a full sternotomy, should hemodynamics intraoperatively deteriorate or if the surgical exposure is suboptimal. A manubriotomy alone also results in improved sternal stability as compared to a full median sternotomy with potentially reduced postoperative patient discomfort. Moreover, upper partial sternal split has an obvious merit from cosmetic perspective with a far shorter incision than median sternotomy. Upper partial sternal split also offers a clear view for both the operator and assistant, in sharp contrast to thoracotomy. This inevitably correlates with the success and safety of the procedure. MICS through a right axillary thoracotomy in pediatric patients can make it difficult for assistants to visualize the detailed cardiac anatomy, which may impair their ability to assist, even during intraoperative complications. In contrast, MICS with UPSS allows the surgeon and assistant to share the same view and respond immediately to intraoperative issues. In addition, MICS with UPSS is well-visualized by assistants during surgery, facilitating instruction of pediatric cardiac fellows during case sharing and making it easier to learn than MICS through a right thoracotomy approach. Although it may initially be challenging, UPSS is a method that anyone can learn, as it is possible to quickly switch to full sternotomy and respond to intraoperative issues.

The only disadvantage is that being a minimally invasive procedure, the upper partial sternal split is definitely technically more challenging, where surgeons need to operate in a limited space with already tight working area in pediatric patients. However, this becomes less challenging and reproducible with experience.

Surgeries for subaortic membrane resection, doubly committed subarterial type VSD, and vascular ring are feasible indications for UPSS if the case does not require re-sternotomy. In the case of aortic arch, UPSS could be considered if the shape and size of aortic arch are suitable for end-to-side anastomosis. In the case of aortic valve stenosis, this method can be used if the disease is unlikely to require conversion to Ross procedure, Konno procedure, or the root replacement.

### Vascular ring

Approaches for vascular ring repair include median sternotomy, thoracotomy, and video-assisted thoracoscopic surgery (VATS). Thoracotomy is the preferred approach in many centers [11, 12]. A good surgical view of the intrathoracic course of the aberrant SCA is achieved through a thoracotomy. However, this method often requires children to be kept on single-lung ventilation during the procedure. In contrast, using a midline approach allows the patient to remain fully ventilated which is beneficial for patients with severe respiratory symptoms [13]. Herrin et al. reported their experience of 115 vascular ring repairs using VATS in Boston Children’s Hospital with excellent long-term results. The advantages of VATS included shorter operation time and hospital stay compared to thoracotomy [5]. Patients who were operated on using VATS were older and heavier (median age and weight: 2.7 years and 14.2 kg) than those operated on through a thoracotomy (median age and weight: 0.9 years and 8.9 kg). In our experience, the upper partial sternal split can be used even in small children. In our series, the youngest patient was 47 days and the lowest weight was 3.1 kg.

With the VATS approach, it can be difficult to reimplant an aberrant SCA to the CCA. No patients underwent reimplantation of SCA via the VATS approach in Herrin’s study and this would seem to corroborate our observation. As stated by Backer et al., division of the ligamentum alone is not adequate for the repair of complex vascular rings. Reimplantation avoids potential left upper limb ischemia and subclavian steal syndrome later in life [14]. The advantage of our approach is that the resection of Kommerell diverticulum and translocation of left SCA to left CCA can be performed with an excellent surgical view.

Backer et al. also recommended complete resection of the Kommerell diverticulum in cases where it is more than 1.5 times the size of the distal SCA, to avoid compression of the esophagus and trachea as well as reduce the chances of aneurysmal rupture or dissection later in life [12]. Furthermore, Luciano et al. found cystic medial necrosis from resected Kommerell diverticulum walls, in at least 50% of specimens, even in very young children. This further reinforces the need to resect the diverticulum during the initial operation [13, 15, 16].

This technique also provides with the option of performing aortopexy if there is vascular compression of the airway as guided by intraoperative bronchoscopy.

### Aortic arch repair with beating heart

To avoid potential tracheal or left pulmonary artery compression, we routinely dissect and mobilize the descending aorta to the level of highest intercostal branches [17]. The view of the surgical field is not different, when compared to a conventional median sternotomy, in terms of recognizing structures around the descending aorta, such as the recurrent laryngeal nerve and left phrenic nerve. We keep the heart beating during aortic cross clamping by perfusing the coronary arteries through an aortic root cannula. We prefer to advance the arterial cannula into the innominate artery for selective antegrade cerebral perfusion and have not found it necessary to suture a Gore-Tex shunt to the innominate artery for arterial cannulation.

### Cosmetic benefits of upper partial sternal split

A review by Konstantinov et al. on minimally invasive procedures in children emphasized the importance of cosmetically superior incisions, especially in the pediatric population, provided that it does not affect patient safety [18]. Observed cosmetic benefits included a smaller incision length, reduced overall wound healing time, and a smaller scar. A study on mini-sternotomy procedures by Vieites et al. also reported improved cosmetic outcomes as well as patient satisfaction, indicating that the perceived benefits certainly manifest in the patients undergoing these operations [19]. The key to ensuring a clear view during surgery is to use chest retractors to spread the wound open in a crisscross pattern, as shown in Fig. 3b. This allows for a wider view without damaging the wound. This makes it possible to apply the UPSS method to the aortic arch repair with end-to-side anastomosis.

## Conclusion

An upper partial sternal split approach is straightforward and can be performed safely with good surgical outcomes and a preferable cosmetic result in selected pediatric cardiac operations.

### Supplementary Information

Below is the link to the electronic supplementary material.

Supplementary file1 (MOV 61358 KB)
